# Synthesis and Antiproliferative Activities of Benzimidazole-Based Sulfide and Sulfoxide Derivatives

**DOI:** 10.3797/scipharm.1507-02

**Published:** 2015-08-18

**Authors:** Samir T. Gaballah, Ahmed O. H. El-Nezhawy, Hassan Amer, Mamdouh Moawad Ali, Abeer Essam El-Din Mahmoud, Andreas Hofinger-Horvath

**Affiliations:** 1 Photochemistry Department, Division of Chemical Industries, National Research Centre, El Buhoth St., Dokki 12622, Giza, Egypt; 2 Department of Pharmaceutical Chemistry, College of Pharmacy, Taif University, Saudi Arabia; 3 Department of Chemistry of Natural and Microbial Products, National Research Centre, El Buhoth St., Dokki 12622, Giza, Egypt; 4 Department of Chemistry, University of Natural Resources and Life Sciences, UFT Campus Tulln, Konrad-Lorenz-Straße 24, A-3430 Tulln, Austria; 5 Biochemistry Department, Division of Genetic Engineering and Biotechnology, National Research Centre, El Buhoth St., Dokki 12622, Giza, Egypt; 6 Department of Chemistry, University of Natural Resources and Life Sciences, Muthgasse 18, A-1190 Vienna, Austria

**Keywords:** Benzimidazole, Sulfide, Sulfoxide, Chemoselective Oxidation, Antiproliferative Activity

## Abstract

The design, synthesis, and *in vitro* antiproliferative activity of a novel series of sulfide (**4a–i**) and sulfoxide (**5a–h**) derivatives of benzimidazole, in which different aromatic and heteroaromatic acetamides are linked to benzimidazole via sulfide (**4a–i**) and sulfoxide (**5a–h**) linker, are reported and the structure-activity relationship is discussed. The new derivatives were prepared by coupling 2-(mercaptomethyl)benzimidazole with 2-bromo-*N*-(substituted) acetamides in dry acetone in the presence of anhydrous potassium carbonate. With very few exceptions, all of the synthesized compounds showed varying antiprolific activities against HepG2, MCF-7, and A549 cell lines. Compound **5a** was very similar in potency to doxorubicin as an anticancer drug, with IC_50_ values 4.1 ± 0.5, 4.1 ± 0.5, and 5.0 ± 0.6 µg/mL versus 4.2 ± 0.5, 4.9 ± 0.6, and 6.1 ± 0.6 µg/mL against HepG2, MCF-7, and A549 cell lines, respectively. In contrast, none of the compounds showed activity against human prostate PC3 cancer cells. Additionally, the sulfoxide derivatives were more potent than the corresponding sulfides.

## Introduction

The high mortality caused by cancer puts it as the number one cause of death worldwide. This represents a great impact on human health, society, and the global economy. Taking into consideration the commercially available cancer therapies, chemotherapy has turned out to be one of the most significant treatments in cancer management [1]. Although a large number of potent chemotherapeutic anticancer agents has been successfully identified, clinical treatments still suffer from many toxic side effects of the drugs such as bone marrow suppression, gastrointestinal tract lesions, nausea, hair loss, drug resistance, and so on [2]. Therefore, the development of novel, efficient, and less toxic anticancer agents which selectively kill or inhibit cell growth of neoplastic cells without affecting non-cancerous host tissues is still of utmost importance.

Benzimidazole derivatives are commonly used chemical scaffolds because they play an important role in medicinal chemistry. They have earned an essential place in the list of chemotherapeutic agents. During the past 5–10 years, many condensation products of benzimidazole have been reported for a variety of biological activities, such as anti-inflammatory [3–5], antiviral [6, 7], and antifungal [8, 9]. Extensive biochemical and pharmacological studies have confirmed that benzimidazoles are effective against various strains of microorganisms [10, 11]. Importantly, the synthesis of benzimidazoles has received much attention due to their antitumor and antiproliferative activities [12–14].

**Fig. 1:**
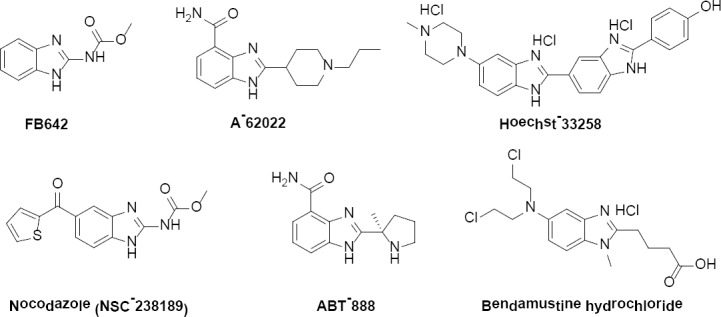
Chemical structure of benzimidazole conjugated with other aliphatic, aromatic, or heterocyclic moieties with pronounced antitumor profiles.

Benzimidazole conjugated with other aliphatic, aromatic, or heterocyclic moieties have resulted in compounds with pronounced antitumor profiles. The chemical structures of some of these conjugates are displayed in Fig. 1. Some of these compounds are in preclinical testing while others are still in the early laboratory investigation phase, for example, FB642 [15–17], A-62022 [18], Hoechst-33258 [19, 20], Nocodazole (NSC-238189) [21, 22], and ABT-888 (Veliparib). The latter is a potential antitumor drug. It inhibits poly(ADP-ribose) polymerase (PARP)-1 and -2, thereby inhibiting DNA repair and potentiating the cytotoxicity of DNA-damaging agents [23–26]. On June 26^th^ 2014, AbbVie announced the initiation of a Phase III clinical trial evaluating the safety and efficacy in patients with advanced breast cancer. Bendamustine hydrochloride is a benzimidazole-based antitumor drug and is being marketed under the commercial name Treanda. It is made by Cephalon Inc. (Frazer, PA. USA). It is an alkylating drug indicated for the treatment of patients with chronic lymphocytic leukemia (CLL), a slowly progressing blood and bone marrow disease [27–30].

As a contribution to this field and in continuation of our previous work on the synthesis and evaluation of new compounds as anticancer agents [31–33], we report herein the synthesis, spectroscopic identification, and *in vitro* antitumor activities of two novel series of benzimidazole-methyl sulfide and benzimidazole-methyl sulfoxide conjugated with aromatic and heteroaromatic acetamide moieties. Data on their antitumor properties is also presented.

## Results and Discussion

### Chemistry

Initially, we focused on the preparation of compounds **3a–i** as previously described in the literature [34–41], starting with the corresponding amine and bromoacetyl bromide in the presence of a base such as triethylamine or potassium carbonate in dry solvent such as dichloromethane (DCM), tetrahydrofuran (THF), or *N,N*-dimethylformamide (DMF) at room temperature. However, the obtained yields were low, which could be attributed to the instability of bromoacetyl bromide due to its rapid decomposition and/or the possibility of the double alkylation of the starting amine under the basic reaction conditions. Consequently, to avoid these difficulties, we prepared compounds **3a–i** by reacting the appropriate amine (**1a–i**) with bromoacetic acid (**2**) in the presence of *N,N*-dicyclohexylcarbodiimide (DCC) in dry THF or DMF with stirring at room temperature to afford compounds **3a–i** in high yields (Sch. 1). The purity of compounds **3a-i** was checked by TLC and their melting points were in good agreement with the literature.

**Sch. 1:**
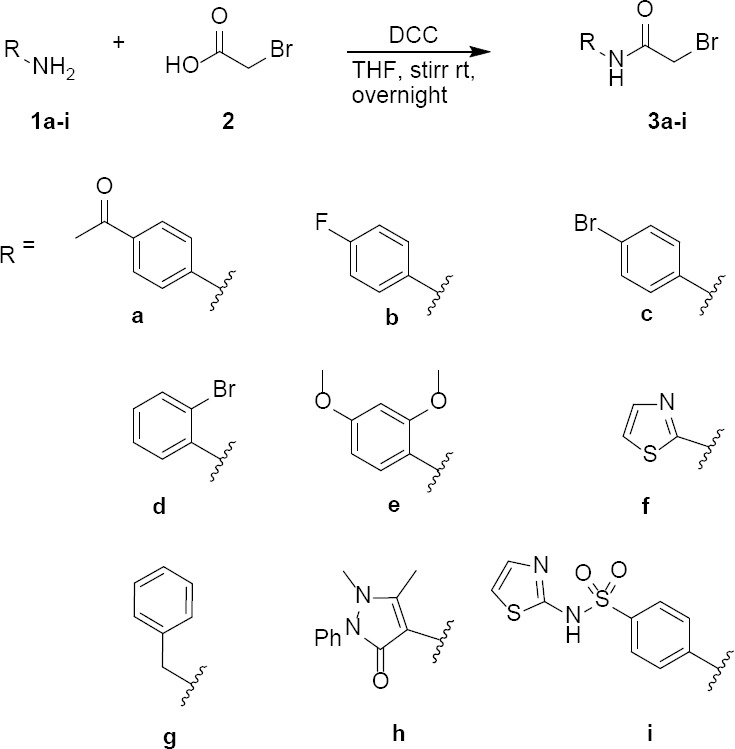
Synthesis of 2-bromo-*N*-substituted acetamides.

Next, 2-(mercaptomethyl)benzimidazole (**BISH**) was prepared according to an adaptation of the Phillips method [42]. Acid-catalyzed condensation of *o*-phenylenediamine with thioglycolic acid at reflux temperature in 4 N HCl afforded the key product after basifying it with ammonium hydroxide. Compound **BISH** was coupled with **3a–i** in acetone in the presence of finely powdered anhydrous potassium carbonate at room temperature to furnish compounds **4a–i** in good yields, (Sch. 2).

**Sch. 2:**
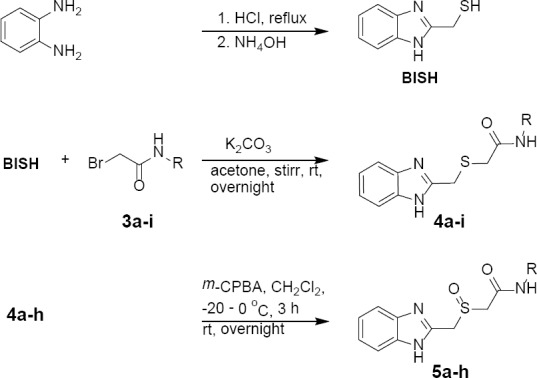
Synthesis of benzimidazole-methyl sulfide and benzimidazole-methyl sulfoxide conjugated with aromatic and heteroaromatic acetamides.

The ^1^H-NMR spectra of **4a–i** were characterized by three distinct and common proton types: (1) the aliphatic protons of the linker which showed two singlets appearing around δ 3.3 and 4.1 ppm assigned to acetamido-methylene and benzimidazolyl-methylene, respectively; (2) the aromatic protons appearing at δ 6.2–7.8 ppm; and (3) two D_2_O-exchangeable protons appearing at about δ 5.6 and < 8.5 ppm assigned to the benzimidazole NH and the amide NH, respectively.

Finally, the target sulfoxides (**5a–h**) were obtained by chemoselective oxidation of the corresponding sulfides **4a–h** (Sch. 2). Many methods have been reported in the literature for the synthesis of sulfoxides [43]. However, double oxidation of the sulfides to sulfones can be a significant issue. Therefore, the reaction conditions such as the molar ratio of the oxidizing agents, temperature, and time must be controlled to prevent over-oxidation. Some commercially available oxidizing agents are inexpensive and efficient. For instance, hydrogen peroxide in the presence of vanadium compounds [44], potassium hydrogen persulfate (oxone) [45], periodate, persulfate, and permanganate are all inorganic oxidizing reagents. However, handling our substrates under aqueous conditions was a major problem. On the other hand, *m*-chloroperbenzoic acid (*m*-CPBA) appeared to be an excellent oxidizing agent with high chemoselectivity. Compounds (**4a–h**) were transformed smoothly to sulfoxide (**5a–h**) analogues using *m*-CPBA in dry dichloromethane at −20 °C. The reaction was stirred further at room temperature to effect the transformation. The transformation of **4i** failed due to its insolubility under the reaction conditions.

The structures of all novel compounds were confirmed by ^1^H- and ^13^C-NMR spectra, mass spectrometry, and microanalyses techniques. Complete and unambiguous assignments for all ^1^H and ^13^C resonances were achieved on the basis of chemical shift considerations and *J*-coupling information. Interestingly, in the ^1^H-NMR spectra of sulfoxides (**5a–h**), the chemical shifts and the coupling constants of the methylene protons of the linker showed a completely different pattern from the one appeared for the corresponding sulfides (**4a–h**). In the sulfoxide, each proton showed a doublet with the coupling constant *J* = 13.4 Hz and the chemical shift was shifted downfield. This could be attributed to the diastereotopic nature of the linker protons affected by the pyramidal chiral sulfoxide group [46. 47]. Also, the chemical shifts of the benzimidazole NH protons (δ 5.6 ppm) for **4a–i** were also moved to the downfield region (δ 9.5 ppm), whereas the amide proton showed a broad singlet around δ 12.5 ppm for compounds **5a–h**.

### Biology

#### Antiproliferative Activity

The antiproliferative activities were expressed by the median growth inhibitory concentration (IC_50_). As shown in Tab. 1, the antiproliferative activities of the synthesized compounds were evaluated against human liver HepG2, breast MCF-7, lung A549, and prostate PC3 cancer cell lines using the sulforhodamine B stain (SRB) assay, in comparison with doxorubicin as a reference drug.

**Tab. 1:**
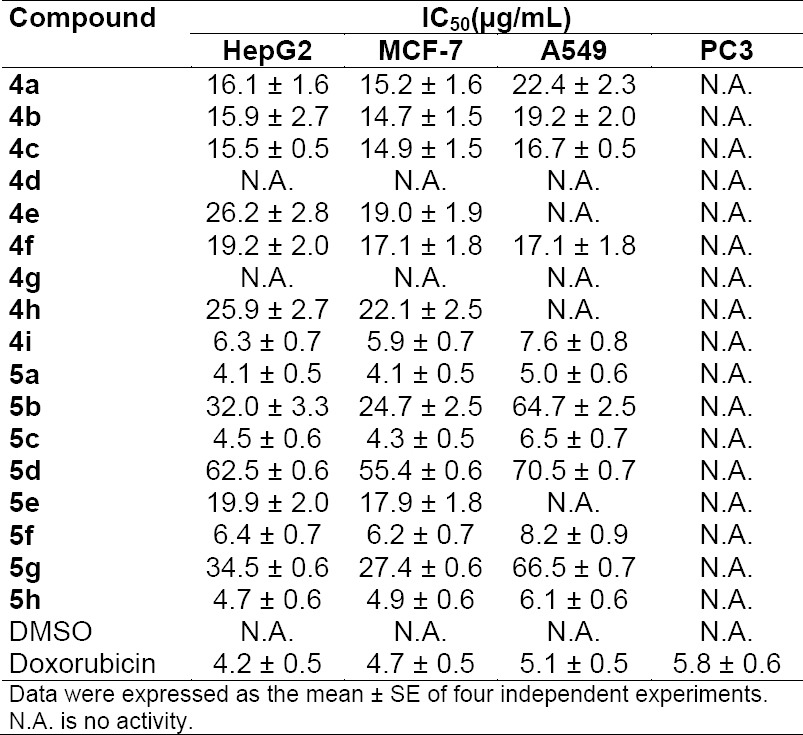
*In vitro* cytotoxicity activity of the tested compounds as expressed as IC_50_ values in 4 human cancer cell lines

None of the compounds exerted any activity against human prostate PC3 cancer cells. The tumor cell line showed normal growth in our culture system and DMSO did not seem to have any noticeable effect on cellular growth. A gradual decrease in viability of cancer cells was observed with increasing concentration of the tested compounds in a dose-dependent inhibitory effect.

For HepG2, MCF-7, and A549 cancer cells, while compounds **4d** and **4g** had no effect on all cancer cells, compound **5a** was similar in potency to doxorubicin as an anticancer drug with an IC_50_ value 4.1 ± 0.5 µg/mL versus 4.2 ± 0.5 µg/mL for doxorubicin against HepG2, 4.1 ± 0.5 µg/mL versus 4.7 ± 0.5 µg/mL for doxorubicin against MCF-7, and 5.0 ± 0.6 µg/mL versus 5.1 ± 0.5 µg/mL for doxorubicin against A549. On the other hand, compounds **4i**, **5a**, **5h**, **5f**, and **5c** were found to be potent anticancer agents and they had IC_50_ values comparable to the standard drug, respectively, 6.3 ± 0.7, 4.1 ± 0.5, 4.7 ± 0.6, 6.4 ± 0.7, and 4.5 ± 0.6 µg/mL versus 4.2 ± 0.5 µg/mL for doxorubicin against the HepG2 cancer cell line. The IC_50_ in the case of MCF-7 cancer cells were, respectively, 5.9 ± 0.7, 4.1 ± 0.5, 4.9 ± 0.6, 6.2 ± 0.7, and 4.3 ± 0.5 µg/mL versus 4.7 ± 0.5 µg/mL for doxorubicin. In the same sense, A549 cells revealed, respectively, IC_50_ values of 7.6 ± 0.8, 5.0 ± 0.6, 6.1 ± 0.6, 8.2 ± 0.9, and 6.5 ± 0.7 µg/mL versus 5.1 ± 0.5 µg/mL for doxorubicin, whereas the rest of compounds had little anticancer activity.

### SAR Analysis

The structure-activity relationship (SAR) investigation of the compounds used in this study gives an understanding of the essential structural requirements for boosting the antiproliferative activities of this class of compounds. The data in Tab. 1 revealed some significant observations: (1) it is noticed that the sulfoxides (**5a–h**) were more potent than the sulfides (**4a–h**) towards all cell lines with **4h** as an exception. (2) The significantly high potency of the latter compound could be attributed to the polar nature of the sulfonamide group as well as the heterocyclic thiazole ring which contributes to the antiprolific effect. (3) Also, the nature of the *N*-substituent on the acetamide was found to affect the activity of these compounds. The heterocyclic substituent had a high impact on the potency as shown by the thiazole, antipyrine, and sulfathiazole moieties, as in, **5f**, **5h**, and **4i**, respectively. (4) The high potency of the *p*-substituted phenyl groups in **5a** and **5c** compared to **5b** could be referred to the polarity and the size of the acetyl and the bromo compared to the fluoro substituent. (5) The substituent in the *m*-position in **4d**, **4e**, **5d**, and **5d** as well as the benzyl group contributes negatively to the antiprolific effect of these compounds. This information on SAR explored in the present study could be helpful in further structural modification and development of new benzimidazole-acetamide hybrids as potent antitumor agents.

## Conclusion

A novel series of sulfide (**4a–i**) and sulfoxide (**5a–h**) derivatives of benzimidazole were synthesized. The synthesized compounds were tested against four different tumor cell lines. The tested compounds exerted antitumor activity in liver HepG2, breast MCF-7, and lung A549 cancer cell lines by reducing cell proliferation and resulted in significant growth inhibitory. Also, the present study revealed that MCF-7 cells were more sensitive to the tested compounds than both HepG2 and A549 cancer cells.

## Experimental

All chemicals were purchased from common commercial suppliers and used without further purification. Melting points (m.p.) were determined on a Gallenkamp Melting Point Apparatus and were uncorrected. ^1^H- and ^13^C-NMR spectra were recorded on a Jeol EX-Spectrometer at 500 and 125 MHz, respectively, in DMSO-*d*_6_ as a solvent at the National Research Centre. Mass spectra were recorded on the Thermo Finnigan SSQ 7000 Advantage spectrometer in EI ionization mode. Microanalyses were performed at the Microanalytical Center in Cairo University. All reactions were performed in air. The reaction progress was monitored using thin-layer chromatography (TLC) which was performed on silica gel 60 F254 aluminum plates (E. Merck, layer thickness 0.2 mm). 4-Amino-*N*-(thiazol-2-yl)benzenesulfonamide (**1i**) was prepared according to published procedures [48, 49].

### Synthesis of 2-Bromo-N-{4-[(1,3-thiazol-2-yl)sulfamoyl]phenyl}acetamide (3i)

Method A: To a mixture of **1i** (2.5 g, 10.0 mmol) and bromoacetic acid (1.7 g, 12.0 mmol) in DMF (20 mL) was added a solution of DCC (2.5, 12.0 mmol) in DMF (5 mL) dropwise with stirring. The reaction mixture was stirred overnight at room temperature. *N,N*-Dicyclohexylurea was filtered off, the filtrate was poured over ice water, and the produced precipitate was collected by filtration.

Method B: To the mixture of **1i** (2.5 g, 10.0 mmol) and triethylamine (1.4 mL, 11.0 mmol) in THF (20 mL) cooled at −20°C was added a solution of bromoacetyl bromide in THF (5 mL) over 30 min. The ice bath was removed after the addition and the reaction mixture was stirred overnight at room temperature. The reaction mixture was concentrated on a rotary evaporator, resuspended in ethyl acetate (50 mL), and then filtered through a pad of silica gel. The filtrate was then concentrated to provide the product as pale yellow powder. Yield: 2.76 g (73%); m.p. 174-176°C; ^1^H-NMR (DMSO-*d*_6_) δ: 3.77 (s, 2H, CH_2_), 6.52 (d, *J* = 2.1 Hz, 1H, aromatic), 6.94 (d, *J* = 2.1, 1H, aromatic), 7.56 (d, *J* = 8.8 Hz, 2H, aromatic), 7.74 (d, *J* = 8.8 Hz, 2H, aromatic), 9.97 (s, 1H, NH), 11.35 (s, 1H, NH) ppm; ^13^C-NMR (DMSO-*d*_6_) δ: 55.99, 102.25, 105.07, 117.43, 126.72, 135.75, 137.27, 144.29, 154.55, 160.56 ppm; Anal. calcd for C_11_H_10_BrN_3_O_3_S_2_ (376.25): C, 35.11; H, 2.68; N, 11.17. Found: C, 34.92; H, 2.89; N, 11.36%.

### General procedure for the synthesis of 2-{[(1H-benzo[d]imidazol-2-yl)methyl]thio}-N-substituted-acetamides (4a–i)

In a 100-mL Erlenmeyer flask with a standard ground top was successively added **BISH** (328 mg, 2 mmol), 2-bromo-*N*-substituted-acetamide (2 mmol), finely ground K_2_CO_3_ (552 mg, 4 mmol), and 15 mL dry acetone. The closed reaction vessel was set at room temperature while the reactants were allowed to stir overnight. After the reaction was complete (inspected by TLC), the vessel content was poured onto crushed ice water and the mixture was stirred for 30 min. The crude product was filtered over a sintered-glass Buchner funnel (porosity grade 4) and the product was washed with cold ether, then hot ether, and dried in air.

#### N-(4-Acetylphenyl)-2-{[(1H-benzimidazol-2-yl)methyl]sulfanyl}acetamide (4a)

Compound **4a** was synthesized according to the above-mentioned general procedure. Yellow powder; Yield 550 mg (81%); m.p. 206–207°C; R_f_ = 0.54 (hexane/ethyl acetate 1:1); ^1^H-NMR (DMSO-*d*_6_) δ: 2.53 (s, 3H, CH_3_CO), 3.49 (s, 2H, CH_2_), 4.08 (s, 2H, CH_2_), 5.56 (s, br, 1H, NH, benzimidazole, D_2_O exchangeable), 7.15-7.16 (m, 2H, aromatic), 7.47–7.55 (m, 2H, aromatic), 7.71–7.72 (m, 2H, aromatic), 7.93–7.94 (m, 2H, aromatic), 10.55 (s, 1H, NH, amide, D_2_O exchangeable) ppm; ^13^C-NMR (DMSO-*d*_6_) δ: 26.38, 28.74, 35.73, 118.73, 129.42, 131.82, 143.18, 151.38, 167.99, 196.42 ppm; Anal calcd for C_18_H_17_N_3_O_2_S (339.41): C, 63.70; H, 5.05; N, 12.38. Found: C, 63.56; H, 5.14; N, 12.59%.

#### 2-{[(1H-Benzimidazol-2-yl)methyl]sulfanyl}-N-(4-fluorophenyl)acetamide (4b)

Compound **4b** was synthesized according to the above-mentioned general procedure. Yellow powder; Yield 550 mg (87%); m.p. 184–185°C; R_f_ = 0.52 (DCM/MeOH; 9.5:0.5); ^1^H-NMR (DMSO-*d*_6_) δ: 3.44 (s, 2H, CH_2_), 4.07 (s, 2H, CH_2_), 5.56 (d, *J* = 7.9 Hz, 1H, NH, benzimidazole, D_2_O exchangeable), 7.13-7.17 (m, 4H, aromatic), 7.50-7.52 (m, 2H, aromatic), 7.59 (dd, 2H, *J* = 8.8, 5.0 Hz, aromatic), 10.26 (s, 1H, NH, amide, D_2_O exchangeable) ppm; ^13^C-NMR (DMSO-*d*_6_) δ: 28.76, 35.57, 115.17, 115.31, 120.88, 120.93, 135.25, 151.44, 156.55, 164.80, 167.29 ppm; Anal calcd for C_16_H_14_FN_3_OS (315.36): C, 60.94; H, 4.47; N, 13.32. Found: C, 60.73; H, 4.59; N, 13.44%.

#### 2-{[(1H-Benzimidazol-2-yl)methyl]sulfanyl}-N-(4-bromophenyl)acetamide (4c)

Compound **4c** was synthesized according to the above-mentioned general procedure. Pale yellow powder; Yield, 600 mg (80%); m.p. 179–181°C; R_f_ = 0.54 (DCM/MeOH; 9.5:0.5); ^1^H-NMR (DMSO-*d*_6_) δ: 3.45 (s, 2H, CH_2_), 4.08 (s, 2H, CH_2_), 5.56 (d, *J* = 8.1 Hz, 1H, NH, benzimidazole, D_2_O exchangeable), 7.15-7.17 (m, 2H, aromatic), 7.48-7.49 (m, 2H, aromatic), 7.50-7.52 (m, 2H, aromatic), 7.54-7.57 (m, 2H, aromatic), 10.34 (s, 1H, NH, amide, D_2_O exchangeable) ppm; ^13^C-NMR (DMSO-*d*_6_) δ: 28.68, 35.66, 114.93, 121.05, 121.73, 131.50, 138.22, 151.41, 167.56 ppm; Anal calcd for C_16_H_14_BrN_3_OS (376.27): C, 51.07; H, 3.75; N, 11.17. Found: C, 50.89; H, 3.56; N, 10.95%.

#### 2-{[(1H-Benzimidazol-2-yl)methyl]sulfanyl}-N-(2-bromophenyl)acetamide (4d)

Compound **4d** was synthesized according to the above-mentioned general procedure. Yellow powder; m.p. 111–114°C; Yield 525 mg (70%); R_f_ = 0.57 (DCM/MeOH; 9.5:0.5); ^1^H-NMR (DMSO-*d*_6_) δ: 3.52 (s, 2H, CH_2_), 4.10 (s, 2H, CH_2_), 5.59 (d, *J* = 7.9 Hz, 1H, NH, benzimidazole, D_2_O exchangeable), 7.13-7.16 (m, 2H, aromatic), 7.28-7.31 (m, 2H, aromatic), 7.47-7.51 (m, 2H, aromatic), 7.65-7.67 (m, 2H, aromatic), 9.86 (s, 1H, NH, amide, D_2_O exchangeable) ppm; ^13^C-NMR (DMSO-*d*_6_) δ: 29.20, 35.64, 117.59, 122.22, 126.70, 127.42, 128.54, 133.17, 136.45, 149.10, 151.82, 157.15, 168.16 ppm; Anal calcd for C_16_H_14_BrN_3_OS (376.27): C, 51.07; H, 3.75; N, 11.17. Found: C, 50.96; H, 3.61; N, 11.02%.

#### 2-{[(1H-Benzimidazol-2-yl)methyl]sulfanyl}-N-(2,4-dimethoxyphenyl)acetamide (4e)

Compound **4e** was synthesized according to the above-mentioned general procedure. Brownish yellow sticky solid; m.p. 105–107°C; Yield 550 mg (77%); R_f_ = 0.47 (DCM/MeOH; 9.5:0.5); ^1^H-NMR (DMSO-*d*_6_) δ: 3.42 (s, 2H, CH_2_), 3.74 (s, 3H, OCH_3_), 3.80 (s, 3H, OCH_3_), 4.07 (s, 2H, CH_2_), 5.57 (s, br, 1H, NH, benzimidazole), 6.47 (d, *J* = 2.7 Hz, 1H, aromatic), 6.61-6.63 (m, 1H, aromatic), 7.05 -7.15 (m, 2H, aromatic), 7.45-7.47 (m, 1H, aromatic), 7.55-7.57 (m, 1H, aromatic), 7.75-7.77 (m, 1H, aromatic), 9.45 (s, 1H, NH, amide) ppm; ^13^C-NMR (DMSO-*d*_6_) δ: 29.07, 35.53, 55.81, 56.27, 99.27, 104.53, 120.71, 122.61, 123.58, 137.30, 137.87, 152.69, 151.93, 157.31, 167.69 ppm; Anal calcd for C_18_H_19_N_3_O_3_S (357.43): C, 60.49; H, 5.36; N, 11.76. Found: C, 60.28; H, 5.49; N, 11.58%.

#### 2-{[(1H-Benzimidazol-2-yl)methyl]sulfanyl}-N-(1,3-thiazol-2-yl)acetamide (4f)

Compound **4f** was synthesized according to the above-mentioned general procedure. Yellow powder; m.p. 218–220°C; Yield 415 mg (68%); R_f_ = 0.49 (DCM/MeOH; 9.5:0.5); ^1^H-NMR (DMSO-*d*_6_) δ: 3.53 (s, 2H, CH_2_), 4.07 (s, 2H, CH_2_), 5.61 (d, *J* = 8.0 Hz, 1H, NH, benzimidazole, D_2_O exchangeable), 7.09 (d, *J* = 3.6 Hz, 1H, aromatic), 7.13-7.15 (m, 2H, aromatic), 7.45 (d, *J* = 3.6 Hz, 1H, aromatic), 7.55-7.56 (m, 2H, aromatic), 9.02 (s, br, 1H, NH, amide, D_2_O exchangeable) ppm; ^13^C-NMR (DMSO-*d*_6_) δ: 29.43, 35.82, 112.46, 114.88, 115.92, 121.37, 137.42, 152.07, 169.02, 168.85 ppm; Anal calcd for C_13_H_12_N_4_OS_2_ (304.39): C, 51.30; H, 3.97; N, 18.41. Found: C, 51.11; H, 3.78; N, 18.19%.

#### 2-{[(1H-Benzimidazol-2-yl)methyl]sulfanyl}-N-benzylacetamide (4g)

Compound **4g** was synthesized according to the above-mentioned general procedure. Pale yellow powder; m.p. 119–122°C; Yield 530 mg (85%); R_f_ = 0.65 (hexane/dichloromethane, 1:1); ^1^H-NMR (DMSO-*d*_6_) δ: 3.31 (s, 2H, CH_2_), 4.04 (s, 2H, CH_2_), 4.29 (d, *J* = 5.8 Hz, 2H, CH_2_), 5.75 (d, *J* = 8.0 Hz, 1H, benzimidazole, D_2_O exchangeable), 7.14-7.17 (m, 2H, aromatic), 7.22-7.25 (m, 1H, aromatic), 7.26-7.28 (m, 2H, aromatic), 7.30-7.33 (m, 2H, aromatic), 7.48-7.51 (m, 2H, aromatic), 8.61 (t, *J* = 5.7 Hz, 1H, NH, amide, D_2_O exchangeable) ppm; ^13^C-NMR (DMSO-*d*_6_) δ: 28.91, 34.61, 42.34, 126.77, 127.20, 128.24, 139.11, 151.55, 168.55 ppm; Anal Calcd for C_17_H_17_N_3_OS (311.40): C, 65.57; H, 5.50; N, 13.49. Found: C, 65.39; H, 5.67; N, 13.32%.

#### 2-{[(1H-Benzimidazol-2-yl)methyl]sulfanyl}-N-(1,5-dimethyl-3-oxo-2-phenyl-2,3-dihydro-1H-pyrazol-4-yl)acetamide (4h)

Compound **4h** was synthesized according to the above-mentioned general procedure. Pale yellow powder; m.p. 135–138°C; Yield 640 mg (79%); R_f_ = 0.58 (hexane/MeOH; 9.5:0.5); ^1^H-NMR (DMSO-*d*_6_) δ: 2.13 (s, 3H, CH_3_), 3.06 (s, 3H, CH_3_), 3.41 (s, 2H, CH_2_), 4.11 (s, 2H, CH_2_), 5.56 (d, *J* = 8.1 Hz, 1H, NH, benzimidazole, D_2_O exchangeable), 7.14-7.16 (m, 2H, aromatic), 7.31-7.34 (m, 1H, aromatic), 7.37 (dd, *J* = 8.5, 1.0 Hz, 2H, aromatic), 7.49-7.52 (m, 4H, aromatic), 9.38 (s, 1H, NH, amide, D_2_O exchangeable) ppm; ^13^C-NMR (DMSO-*d*_6_) δ: 11.07, 28.61, 34.40, 35.91, 107.23, 123.52, 126.26, 129.05, 134.92, 151.47, 152.11, 156.57, 161.62, 168.17 ppm; Anal calcd for C_21_H_21_N_5_O_2_S (407.49): C, 61.90; H, 5.19; N, 17.19. Found: C, 62.11; H, 5.36; N, 16.98%.

#### 2-{[(1H-Benzimidazol-2-yl)methyl]sulfanyl}-N-{4-[(1,3-thiazol-2-yl)sulfamoyl]phenyl}acetamide (4i)

Compound **4i** was synthesized according to the above-mentioned general procedure. Pale yellow powder; m.p. 250°C (dec.); Yield 480 mg (52%); R_f_ = 0.33 (DCM/MeOH; 9.5:0.5); ^1^H-NMR (DMSO-*d*_6_) δ: 3.35 (s, b, 1H, D_2_O exchangeable), 3.47 (s, 2H, CH_2_), 4.07 (s, 2H, CH_2_), 5.75 (s, br, 1H, NH, benzimidazole), 6.81 (d, *J* = 4.6 Hz, 1H, aromatic), 7.12-7.15 (m, 2H, aromatic), 7.24 (d, *J* = 4.6 Hz, 1H, aromatic), 7.49-7.50 (m, 2H, aromatic), 7.70-7.71 (m, 2H, aromatic), 7.74-7.76 (m, 2H, aromatic), 10.53 (s, 1H, NH, benzimidazole, D_2_O, exchangeable) ppm; ^13^C-NMR (DMSO-*d*_6_) δ: 28.73, 35.69, 108.05, 118.68, 121.62, 124.43, 126.91, 136.54, 141.99, 151.37, 167.94, 168.66 ppm; Anal calcd for C_19_H_17_N_5_O_3_S_3_ (459.56): C, 49.66; H, 3.73; N, 15.24. Found: C, 49.48; H, 3.90; N, 15.36%.

### General Procedure for the Chemoselective Oxidation of 4a–h

A solution of *m*-CPBA (1.2 mmol) in DCM (10 mL) was added dropwise to **4a–h** (1 mmol) dissolved in DCM (35 mL) cooled to −20°C for 30 min. The reaction mixture was stirred for an additional 2h. The temperature was raised gradually to room temperature and the reaction was stirred overnight. After complete consumption of the starting materials as indicated by TLC, water was added and the organic product was extracted with ethyl acetate. The combined organic extract was collected, dried over anhydrous magnesium sulfate, and the solvent was removed under reduced pressure to afford pure sulfoxide products **5a–h** in high yields.

#### N-(4-Acetylphenyl)-2-[(1H-benzimidazol-2-yl)methanesulfinyl]acetamide (5a)

Compound **5a** was synthesized according to the above-mentioned general procedure. Pale yellow powder; m.p. 122–124°C; Yield 300 mg (84%); R_f_ = 0.37 (ethyl acetate/MeOH; 9:1); ^1^H-NMR (DMSO-*d*_6_) δ: 2.46 (s, 3H, CH_3_CO), 3.89 (d, *J* = 13.5 Hz, 1H, CH_2_), 4.20 (d, *J* = 13.5 Hz, 1H, CH_2_), 4.38 (d, *J* = 13.5 Hz, 1H, CH_2_), 4.58 (d, *J* = 13.5 Hz, 1H, CH_2_), 7.16-7.18 (m, 2H, aromatic), 7.53-7.55 (m, 2H, aromatic), 7.69-7.71 (m, 2H, aromatic), 7.90-7.92 (m, 2H, aromatic), 10.55 (s, 1H, NH), 12.70 (s, 1H, NH) ppm; MS EI m/z (%): 355 [M^+^] (0.03%), 304 (2.5), 255 (4.3), 209 (3.1), 159 (6.0), 155 (15), 138 (23), 135 (52), 132 (81), 120 (100), 92 (29), 77 (11); Anal calcd for C_18_H_17_N_3_O_3_S (355.41): C, 60.83; H, 4.82; N, 11.82. Found: C, 61.01; H, 4.67; N, 12.05%.

#### 2-[(1H-Benzimidazol-2-yl)methanesulfinyl]-N-(4-fluorophenyl)acetamide (5b)

Compound **5b** was synthesized according to the above-mentioned general procedure. Pale yellow powder; m.p. 183–184°C; Yield 310 mg (94%); R_f_ = 0.46 (ethyl acetate/MeOH; 9:1); ^1^H-NMR (DMSO-*d*_6_) δ: 3.83 (d, *J* = 14.35 Hz, 1H, CH_2_), 4.15 (d, *J* = 14.35 Hz, 1H, CH_2_), 4.37 (d, *J* = 14.35 Hz, 1H, CH_2_), 4.57 (d, *J* = 14.35 Hz, 1H, CH_2_), 7.11-7.51 (m, 2H, aromatic), 7.48-7.51 (m, 2H, aromatic), 7.57-7.59 (m, 2H, aromatic), 7.65-7.66 (m, 2H, aromatic), 10.48 (s, 1H, NH), 13.46 (s, br, 1H, NH) ppm; MS EI m/z (%): 331 [M^+^] (0.04%), 269 (1.7), 236 (1.6), 158 (19.0), 157 (6.0), 156 (61), 141 (7.6), 139 (89.4), 132 (8.1), 131 (18.8), 113 (23.5), 112 (9.2), 111(100), 77 (20.1), 76 (20.7), 75 (63.5), 74 (37.8); Anal calcd for C_16_H_14_FN_3_O_2_S (331.36): C, 57.99; H, 4.26; N, 12.68. Found: C, 57.78; H, 4.37; N, 12.85%.

#### 2-[(1H-Benzimidazol-2-yl)methanesulfinyl]-N-(4-bromophenyl)acetamide (5c)

Compound **5c** was synthesized according to the above-mentioned general procedure. Pale yellow; m.p. 217–219°C; Yield 340 mg (87%); R_f_ = 0.17 (ethyl acetate/MeOH; 9:1); ^1^H-NMR (DMSO-*d*_6_) δ: 3.84 (d, *J* = 13.4 Hz, 1H, CH_2_), 4.15 (d, *J* = 13.4 Hz, 1H, CH_2_), 4.36 (d, *J* = 13.4 Hz, 1H, CH_2_), 4.56 (d, *J* = 13.4 Hz, 1H, CH_2_), 7.16-7.17 (m, 2H, aromatic), 7.46-7.49 (m, 3H, aromatic), 7.52-7.55 (m, 3H, aromatic), 10.54 (s, 1H, NH), 12.60 (s, 1H, NH) ppm; MS EI m/z (%): 393 [M^+^ + 1] (0.34), 391 (3.41), 380 (4.62), 332 (1.72), 215 (3.29), 213 (4.60), 199 (4.6), 197 (4.5), 173 (32.1), 171 (35.5), 132 (100.0), 131 (75.0), 92 (29.4), 91 (22.0), 90 (24.2), 65 (36.6), 64 (42.7), 63 (41.5); Anal calcd for C_16_H_14_BrN_3_O_2_S (392.27): C, 48.99; H, 3.60; N, 10.71. Found: C, 48.78; H, 3.76; N, 10.90%.

#### 2-[(1H-Benzimidazol-2-yl)methanesulfinyl]-N-(2-bromophenyl)acetamide (5d)

Compound **5d** was synthesized according to the above-mentioned general procedure. Pale yellow powder; m.p. 158–160°C; Yield 315 mg (80%); R_f_ = 0.56 (ethyl acetate/MeOH; 9:1); ^1^H-NMR (DMSO-*d*_6_) δ: 3.97 (d, *J* = 13.5 Hz, 1H, CH_2_), 4.23 (d, *J* = 13.5 Hz, 1H, CH_2_), 4.37 (d, *J* = 13.5 Hz, 1H, CH_2_), 4.57 (d, *J* = 13.5 Hz, 1H, CH_2_), 7.13-7.16 (m, 3H, aromatic), 7.33-7.35 (m, 1H, aromatic), 7.51-7.61 (m, 3H, aromatic), 7.63-7.65 (m, 1H, aromatic), 10.01 (s, 1H, NH), 12.70 (s, 1H, NH) ppm; ^13^C-NMR (DMSO-*d*_6_) δ: 50.87, 57.59, 115.50, 118.03, 122.57, 127.45, 127.95, 128.46, 128.60, 131.50, 133.30, 136.15, 145.89, 164.17 ppm; MS EI m/z (%): 393 [M^+^ + 1] (0.17), 335 (6.7), 333 (6.5), 173 (48.9), 171 (52.0), 132 (100.0), 131 (79.5), 92 (42.6), 91 (24.3), 90 (22.5), 65 (41.8), 64 (42.3), 63 (31.0); Anal calcd for C_16_H_14_BrN_3_O_2_S (392.27): C, 48.99; H, 3.60; N, 10.71. Found: 49.17; H, 3.73; N, 10.56%.

#### 2-[(1H-Benzimidazol-2-yl)methanesulfinyl]-N-(2,4-dimethoxyphenyl)acetamide (5e)

Compound **5e** was synthesized according to the above-mentioned general procedure. Pale yellow powder; m.p. 180–182°C; Yield 301 mg (81%); R_f_ = 0.17 (ethyl acetate/MeOH; 9:1); ^1^H-NMR (DMSO-*d*_6_) δ: 3.74 (s, 3H, OCH_3_), 3.81 (s, 3H, OCH_3_), 4.0 (d, *J* = 13.4 Hz, 1H, CH_2_), 4.22 (d, *J* = 13.4 Hz, 1H, CH_2_), 4.37 (d, *J* = 13.4, 1H, CH_2_), 4.57 (d, *J* = 13.4 Hz, 1H, CH_2_), 6.5 (dd, *J* = 9.1, 2.4 Hz, 1H, aromatic), 6.63 (d, *J* = 1.9 Hz, 1H, aromatic), 7.2 (dd, *J* = 5.7, 2.9 Hz, 2H, aromatic), 7.57 (s, br, 2H, aromatic), 7.77 (d, *J* = 8.6, 1H, aromatic), 9.62 (s, 1H, NH), 12.65 (s, 1H, NH) ppm; ^13^C-NMR (DMSO-*d*_6_) δ: 50.88, 50.98, 55.77, 57.84, 99.26, 104.59, 120.24, 122.41, 123.97, 146.07, 151.71, 151.79, 157.61, 163.37 ppm; MS EI m/z (%): 376 [M^+^ + 3] (19.7), 345 (9.5), 227 (6.0), 179 (15.0), 164 (14.1), 153 (75.1), 138 (67.0), 132 (100.0), 131 (72.0), 110 (25.8), 95 (18.3), 64 (22.2); Anal calcd for C_18_H_19_N_3_O_4_S (373.43): C, 57.89; H, 5.13; N, 11.25. Found: C, 58.11; H, 5.02; N, 11.07%.

#### 2-[(1H-Benzimidazol-2-yl)methanesulfinyl]-N-(1,3-thiazol-2-yl)acetamide (5f)

Compound **5f** was synthesized according to the above-mentioned general procedure. Yellow powder; m.p. 185–187°C; Yield 240 mg (75%); R_f_ = 0.31 (ethyl acetate/MeOH; 9:1); ^1^H-NMR (DMSO-*d*_6_) δ: 4.01 (d, *J* = 13.5 Hz, 1H, CH_2_), 4.27 (d, *J* = 13.5 Hz, 1H, CH_2_), 4.39 (d, *J* = 13.5 Hz, 1H, CH_2_), 4.58 (d, *J* = 13.5 Hz, 1H, CH_2_), 7.15-7.28 (m, 3H, aromatic), 7.47-7.53 (m, 3H, aromatic), 12.55 (s, br, 2H, 2NH) ppm; MS EI m/z (%): 320 [M^+^] (0.12), 302 (1.8), 256 (3.1), 204 (8.7), 133 (13.3), 132 (100), 131 (99.0), 118 (13.0), 100 (81.0), 64 (24.3), 58 (39.7); Anal calcd for C_13_H_12_N_4_O_2_S_2_ (320.39): C, 48.73; H, 3.78; N, 17.49. Found: C, 48.91; H, 3.62; N, 17.64%.

#### 2-[(1H-Benzimidazol-2-yl)methanesulfinyl]-N-benzylacetamide (5g)

Compound **5g** was synthesized according to the above-mentioned general procedure. Pale yellow powder; m.p. 184–186°C; Yield 295 mg (90%); R_f_ = 0.29 (ethyl acetate/MeOH; 9:1); ^1^H-NMR (DMSO-*d*_6_) δ: 3.70 (d, *J* = 13.4 Hz, 1H, CH_2_), 3.99 (d, *J* = 13.4 Hz, 1H, CH_2_), 4.28-4.34 (m, 1H (CH_2_) and 2H (ph- CH_2_)), 4.51 (d, *J* = 13.4 Hz, 1H, CH_2_), 7.15-7.25 (m, 8H aromatic), 7.26-7.28 (m, 1H, aromatic), 8.84 (s, 1H, NH), 12.57 (s, 1H, NH) ppm; MS EI m/z (%): 327 [M^+^] (0.27), 305 (0.4), 284 (2.8), 255 (1.7), 193 (13.8), 148 (26.7), 133 (30.4), 132 (100), 131 (77.2), 118 (8.4), 106 (36.4), 91 (88.0), 77 (15.5), 64 (20.0); Anal calcd for C_17_H_17_N_3_O_2_S (327.40): C, 62.36; H, 5.23; N, 12.83. Found: C, 62.48; H, 5.09; N, 12.64%.

#### 2-[(1H-Benzimidazol-2-yl)methanesulfinyl]-N-(1,5-dimethyl-3-oxo-2-phenyl-2,3-dihydro-1H-pyrazol-4-yl)acetamide (5h)

Compound **5h** was synthesized according to the above-mentioned general procedure. Pale yellow powder; m.p. 220–222°C; Yield 340 mg (80%); R_f_ = 0.16 (ethyl acetate/MeOH; 8:2). ^1^H-NMR (DMSO-*d*_6_) δ: 2.10 (s, 3H, CH_3_), 2.46 (s, 3H, CH_3_), 3.70 (d, *J* = 14.35 Hz, 1H, CH_2_), 4.11 (d, *J* = 14.35 Hz, 1H, CH_2_), 4.37 (d, *J* = 14.35 Hz, 1H, CH_2_), 4.55 (d, *J* = 14.3 1H, CH_2_), 7.14-7.16 (m, 2H, aromatic), 7.29-7.32 (m, 3H, aromatic), 7.45-7.53 (m, 4H, aromatic), 9.63 (s, 1H, NH), 12.64 (s, 1H, NH) ppm; ^13^C-NMR (DMSO-*d*_6_) δ: 11.78, 36.77, 50.94, 57.27, 107.31, 122.41, 124.17, 126.89, 129.65, 135.41, 146.02, 152.77, 161.96, 164.42 ppm; MS EI m/z (%): 425 [M^+^+2] (0.24), 414 (1.5), 380 (3.9), 256 (2.8), 245 (4.8), 203 (13.6), 180 (6.2), 160 (4.3), 133 (13.0), 132 (100), 131 (77.5), 104 (16.3), 93 (16.6), 84 (35.0), 77 (27.7), 64 (45.4), 56 (83.7); Anal calcd for C_21_H_21_N_5_O_3_S (423.49): C, 59.56; H, 5.00; N, 16.54. Found: C, 59.78; H, 4.87; N, 16.68%.

### Biology

#### Chemicals

Fetal bovine serum (FBS) and L-glutamine were obtained from Gibco Invitrogen Company (Scotland, UK). Dulbecco’s Modified Eagle’s (DMEM) Medium was provided by Cambrex (New Jersey, USA). Dimethyl sulfoxide (DMSO), doxorubicin, penicillin, streptomycin, sulforhodamine B stain (SRB) (3-(4,5-dimethylthiazol-2-yl)-2,5-diphenyltetrazolium bromide), and all other chemicals and reagents used in this study were of analytical grade and purchased from Sigma-Aldrich Chemical Co. (St. Louis, MO, USA).

Anticancer activity screening for the tested compounds utilizing four different human tumor cell lines including liver HepG2, breast MCF-7, lung A549, and prostate PC3 cancer cell lines were obtained from the American Type Culture Collection (Rockville, MD, USA). The tumor cells were maintained in Dulbecco’s Modified Eagle’s Medium (DMEM) supplemented with 10% heat-inactivated fetal calf serum (GIBCO), penicillin (100 U/mL), and streptomycin (100 µg/mL) at 37°C in a humidified atmosphere containing 5% CO_2_. Cells at a concentration of 0.50 × 10^6^ were grown in a 25 cm^2^ flask in 5 mL of complete culture medium.

### In Vitro Cytotoxicity Assay

The antiproliferative activity was measured *in vitro* using the SRB assay according to the previously reported standard procedure [50]. Cells were inoculated in a 96-well microtiter plate (10^4^ cells/well) for 24 h before treatment with the tested compounds to allow attachment of cells to the wall of the plate. The test compounds were dissolved in DMSO at 1 mg/mL immediately before use and diluted to the appropriate volume just before the addition to the cell culture. Different concentrations of test compounds (0–100 µg/mL) and doxorubicin were added to the cells. Four wells were prepared for each individual dose. Monolayer cells were incubated with the compounds for 48 h at 37°C and in an atmosphere of 5% CO_2_. After 48 h cells were fixed, washed, and stained for 30 min with 0.4% (w/v) SRB, then dissolved in 1% acetic acid. Unbound dye was removed by four washes with 1% acetic acid, and the attached stain was recovered with Tris-EDTA buffer. Color intensity was measured in an ELISA reader at wavelength 540 nm. The relation between the surviving fraction and drug concentration was plotted to get the survival curve for each cell line after the specified time. The concentration required for 50% inhibition of cell viability (IC_50_) was calculated and the results are given in Tab. 1.

### Statistical Analysis

The results are reported as the mean ± standard error (S.E.) for at least four experiments.

## References

[1] Harrison M, Holen K, Liu G (2009). Beyond Taxanes: a Review of Novel Agents that Target Mitotic Tubulin and Microtubules, Kinases, and Kinesins. Clin Adv Hematol Oncol.

[2] Nussbaumer S, Bonnabry P, Veuthey J, Fleury-Souverain S (2011). Analysis of Anticancer Drugs:A Review. Talanta.

[3] El-Nezhawy AOH, Gaballah ST, Radwan MAA, Baiuomy AR, Abdel-Salam OME (2009). Structure-Based Design of Benzimidazole Sugar Conjugates: Synthesis, SAR and In Vivo Anti-inflammatory and Analgesic Activities. Med Chem.

[4] Evans D, Hicks TA, Williamson WRN, Dawson W, Meacock SCR, Kitchen EA (1996). Synthesis of a Group of 1H-Benzimidazoles and their Screening for Antiinflammatory Activity. Eur J Med Chem.

[5] Gilman SC, Carlson RP, Chang J, Lewis AJ (1985). The Antiinflammatory Activity of the Immunomodulator Wy18,251 (3-(p-chlorophenyl)thiazolo[3,2-a]benzimidazole-2-acetic acid). Inflammation Res.

[6] Porcari AR, Devivar RV, Kucera LS, Drach JC, Townsend LB (1998). Design, Synthesis, and Antiviral Evaluations of 1-(Substitutedbenzyl)-2-substituted-5,6-dichlorobenzimidazoles as Nonnucleoside Analogues of 2,5,6-Trichloro-1-(a-D-ribofuranosyl)benzimidazole. J Med Chem.

[7] Townsend LB, Devivar RV, Turk SR, Nassiri MR, Drach JC (1996). Design, Synthesis, and Antiviral Activity of Certain 2,5,6-Trihalo-1-b-D-ribofuranosyl)benzimidazoles. J Med Chem.

[8] Poeta MD, Schell WA, Dykstra CC, Jones S, Tidwell RR, Czarny A, Bajic M, Bajic M, Kumar A, Boykin D, Perfect JR (1998). Structure-In Vitro Activity Relationships of Pentamidine Analogues and Dication-Substituted Bis-Benzimidazoles as New Antifungal Agents. Antimicrob Agents Chemother.

[9] Küçükbay H, Durmaz R, Orhan E, Günal S (2003). Synthesis, Antibacterial and Antifungal Activities of Electron-rich Olefins Derived Benzimidazole Compounds. Farmaco.

[10] Yalcin I, Oren I, Sener E, Akin A, Ucarturk N (1992). The Synthesis and the Structure-activity Relationships of some Substituted Benzoxazoles, Oxazolo(4,5-b)pyridines, Benzothiazoles and Benzimidazoles as Antimicrobial Agents. Eur J Med Chem.

[11] Klimešová V, Kočı J, Pour M, Stachel J, Waisser K, Kaustová J (2002). Synthesis and Preliminary Evaluation of Benzimidazole Derivatives as Antimicrobial Agents. Eur J Med Chem.

[12] Settimo AD, Settimo FD, Marini AM, Primofiore G, Salerno S, Viola G, Via LD, Magno SM (1998). Synthesis, DNA Binding and in vitro Antiproliferative Activity of Purinoquinazoline, Pyridopyrimidopurine and Pyridopyrimidobenzimidazole Derivatives as Potential Antitumor Agents. Eur J Med Chem.

[13] Abdel-Mohsen HT, Ragab FAF, Ramla MM, Diwani HIE (2010). Novel Benzimidazole-pyrimidine Conjugates as Potent Antitumor Agents. Eur J Med Chem.

[14] Craigo WA, LeSueur BW, Skibo EB (1999). Design of Highly Active Analogues of the Pyrrolo[1,2-a]benzimidazole Antitumor Agents. J Med Chem.

[15] Hao D, Rizzo JD, Stringer S, Moore RV, Marty J, Dexter DL, Mangold GL, Camden JB, Hoff DDV, Weitman SD (2002). Preclinical Antitumor Activity and Pharmacokinetics of Methyl 2-Benzimidazolecarbamate (FB642). Invest New Drugs.

[16] Hammond LA, Davidson K, Lawrence R, Camden JB, Hoff DDV, Weitman S, Izbicka E (2001). Exploring the Mechanisms of Action of FB642 at the Cellular Level. J Cancer Res Clin Oncol.

[17] Desai NC, Dodiya AM, Shihory NR (2012). A search of Novel Antimicrobial Based on Benzimidazole and 2-Pyridone Heterocycles. Med Chem Res.

[18] Penning TD, Zhu G-D, Gandhi VB, Gong J, Thomas S, Lubisch W, Grandel R, Wernet W, Park CH, Fry EH, Liu X, Shi Y, Klinghofer V, Johnson EF, Donawho CK, Frost DJ, Bontcheva-Diaz V, Bouska JJ, Olson AM, Marsh KC, Luo Y, Rosenberg SH, Giranda VL (2008). Discovery and SAR of 2-(1-Propylpiperidin-4-yl)-1H-benzimidazole-4-carboxamide:A Potent Inhibitor of Poly(ADP-ribose) polymerase (PARP) for the Treatment of Cancer. Bioorg Med Chem.

[19] Gravatt GL, Baguley BC, Wilson WR, Denny WA (1994). DNA-Directed Alkylating Agents. 6. Synthesis and Antitumor Activity of DNA Minor Groove-Targeted Aniline Mustard Analogs of Pibenzimol (Hoechst 33258). J Med Chem.

[20] Singh M, Tandon V (2011). Synthesis and Biological Activity of Novel Inhibitors of Topoisomerase I:2-Aryl-substituted 2-Bis-1H-benzimidazoles. Eur J Med Chem.

[21] Wang Y-J, Jeng J-H, Chen R-J, Tseng H, Chen L-C, Liang Y-C, Lin C-H, Chen C-H, Chu J-S, Ho W-L, Ho Y-S (2002). Ketoconazole Potentiates the Antitumor Effects of Nocodazole: In vivo Therapy for Human Tumor Xenografts in Nude Mice. Mol Carcinog.

[22] Duanmu C, Shahrik LK, Ho HH, Hamel E (1989). Tubulin-dependent Hydrolysis of Guanosine Triphosphate as a Screening Test to Identify New Antitubulin Compounds with Potential as Antimitotic Agents:Application to Carbamates of Aromatic Amines. Cancer Res.

[23] Donawho CK, Luo Y, Penning TD, Bauch JL, Bouska JJ, Bontcheva-Diaz VD, Cox BF, DeWeese TL, Dillehay LE, Ferguson DC, Ghoreishi-Haack NS, Grimm DR, Guan R, Han EK, Holley-Shanks RR, Hristov B, Idler KB, Jarvis K, Johnson EF, Kleinberg LR, Klinghofer V, Lasko LM, Liu X, Marsh KC, McGonigal TP, Meulbroek JA, Olson AM, Palma JP, Rodriguez LE, Shi Y, Stavropoulos JA, Tsurutani AC, Zhu GD, Rosenberg SH, Giranda VL, Frost DJ (2007). ABT-888, an Orally Active Poly(ADP-Ribose) Polymerase Inhibitor that Potentiates DNA-Damaging Agents in Preclinical Tumor Models. Clin Cancer Res.

[24] Albert JM, Cao C, Kim KW, Willey CD, Geng L, Xiao D, Wang H, Sandler A, Johnson DH, Colevas AD, Low J, Rothenberg ML, Lu B (2007). Inhibition of Poly(ADP-Ribose) Polymerase Enhances Cell Death and Improves Tumor Growth Delay in Irradiated Lung Cancer Models. Clin Cancer Res.

[25] Kummar S, Kinders R, Gutierrez ME, Rubinstein L, Parchment RE, Phillips LR, Ji J, Monks A, Low JA, Chen A, Murgo AJ, Collins J, Steinberg SM, Eliopoulos H, Giranda VL, Gordon G, Helman L, Wiltrout R, Tomaszewski JE, Doroshow JH (2009). Phase 0 Clinical Trial of the Poly (ADP-Ribose) Polymerase Inhibitor ABT-888 in Patients With Advanced Malignancies. J Clin Oncol.

[26] Penning TD, Zhu G-D, Gandhi VB, Gong J, Liu X, Shi Y, Klinghofer V, Johnson EF, Donawho CK, Frost DJ, Bontcheva-Diaz V, Bouska JJ, Osterling DJ, Olson AM, Marsh KC, Luo Y, Giranda VL (2009). Discovery of the Poly(ADP-ribose) Polymerase (PARP) Inhibitor 2-[(R)-2-Methylpyrrolidin-2-yl]-1H-benzimidazole-4-carboxamide (ABT-888) for the Treatment of Cancer. J Med Chem.

[27] Kath R, Blumenstengel K, Fricke HJ, Höffken K (2001). Bendamustine Monotherapy in Advanced and Refractory Chronic Lymphocytic Leukemia. J Cancer Res Clin Oncol.

[28] Schwänen C, Hecker T, Hübinger G, Wölfle M, Rittgen W, Bergmann L, Karakas T (2002). In vitro Evaluation of Bendamustine Induced Apoptosis in B-Chronic Lymphocytic Leukemia. Leukemia.

[29] Cheson BD, Wendtner C-M, Pieper A, Dreyling M, Friedberg J, Hoelzer D, Moreau P, Gribben J, Knop S, Montillo M, Rummel M (2010). Optimal Use of Bendamustine in Chronic Lymphocytic Leukemia, Non-Hodgkin Lymphomas, and Multiple Myeloma:Treatment Recommendations From an International Consensus Panel. Clin Lymphoma Myeloma Leuk.

[30] Aivado M, Schulte K, Henze L, Burger J, Haas R (2002). Bendamustine in the Treatment of Chronic Lymphocytic Leukemia:Results and Future Perspectives. Semin Oncol.

[31] El-Nezhawy AOH, Radwan MAA, Gaballah ST (2009). Synthesis of Chiral N-(2-(1-Oxophthalazin-2(1H)-yl)ethanoyl)-α-amino Acid Derivatives as Antitumor Agents. ARKIVOC.

[32] El-Nezhawy AOH, Adly FG, Eweas AF, Hanna AG, El-Kholy YM, El-Sayed SH, El-Naggar TBA (2011). Synthesis of Some Novel D-Glucuronic Acid Acetylated Derivatives as Potential Anti-Tumor Agents. Arch Pharm Chem Life Sci.

[33] El-Nezhawy AOH, Adly FG, Eweas AF, Hanna AG, El-Kholy YM, El-Naggar TBA (2011). Design, Synthesis and Antitumor Activity of Novel D-Glucuronic Acid Derivatives. Med Chem.

[34] Cheung Y-Y, Zamorano R, Blobaum AL, Weaver CD, Conn PJ, Lindsley CW, Niswender CM, Hopkins CR (2011). Solution-Phase Parallel Synthesis and SAR of Homopiperazinyl Analogs as Positive Allosteric Modulators of mGlu4. ACS Comb Sci.

[35] Wang Z, Wu B, Kuhen KL, Bursulaya B, Nguyen TN, Nguyen DG, He Y (2006). Synthesis and Biological Evaluations of Sulfanyltriazoles as Novel HIV1 Non-nucleoside Reverse Transcriptase Inhibitors. Bioorg Med Chem Lett.

[36] Zorn B, Schmidt F (1957). [N-Acyl Derivative of 1-Phenyl-2,3-dimethyl-4-aminopyrazolon-5-(4-aminoantipyrine)]. Pharmazie.

[37] Tabrizi MA, Baraldi PG, Preti D, Romagnoli R, Saponaro G, Baraldi S, Moorman AR, Zaid AN, Varani K, Borea PA (2008). 1,3-Dipropyl-8-(1-phenylacetamide-1H-pyrazol-3-yl)-xanthine Derivatives as Highly Potent and Selective Human A 2B Adenosine Receptor Antagonists. Bioorg Med Chem.

[38] Kirubakaran S, Gorla SK, Sharling L, Zhang M, Liu X, Ray SS, MacPherson IS, Striepen B, Hedstrom L, Cuny GD (2012). Structure-Activity Relationship Study of Selective Benzimidazole-Based Inhibitors of Cryptosporidium Parvum IMPDH. Bioorg Med Chem Lett.

[39] Zhou B, He Y, Zhang X, Xu J, Luo Y, Wang Y, Franzblau SG, Yang Z, Chan RJ, Liu Y, Zheng J, Zhang ZY (2010). Targeting Mycobacterium Protein Tyrosine Phosphatase B for Antituberculosis Agents. Proc Nat Acad Sci U S A.

[40] Torres-Murro J, Quintero L, Sartillo-Piscil F (2005). Regioselective Alkylation of 3,4-Dihydro-2H-pyran by Xanthate-mediated free Radical Nonchain Process. Tetrahedron Lett.

[41] Abuo-Rahma GE-DAA, Abdel-Aziz M, Beshr EAM, Ali TFS (2014). 1,2,4-Triazole/Oxime Hybrids as New Strategy for Nitric Oxide Donors:Synthesis, Anti-inflammatory, Ulceroginicity and Antiproliferative Activities. Eur J Med Chem.

[42] Milner ES, Snyder S, Joullie MM (1964). 795. Synthesis of Benzimidazol-2-ylalkanethiols and Some Derivatives. J Chem Soc.

[43] Madesclaire M (1986). Synthesis of Sulfoxides by Oxidation of Thioethers. Tetrahedron.

[44] Kaczorowska K, Kolarska Z, Mitka K, Kowalski P (2005). Oxidation of Sulfides to Sulfoxides. Part 2:Oxidation by Hydrogen Peroxide. Tetrahedron.

[45] Ali M, Kriedelbaugh D, Wencewicz T (2007). Ceric Ammonium Nitrate Catalyzed Oxidation of Sulfides to Sulfoxides. Synthesis.

[46] Viau R, Durst T (1973). Kinetic Preference between the Diastereotopic Hydrogens in the Lithiation of Benzyl Methyl and Benzyl tert-Butyl Sulfoxides. J Am Chem Soc.

[47] Solladie G, Zimmermann RG (1984). Enantiospecific Synthesis of Optically Active Cyclohexylidene Bromomethanes. Tetrahedron Lett.

[48] Fosbinder RJ, Walter LA (1939). Sulfanilamido Derivatives of Heterocyclic Amines. J Am Chem Soc.

[49] Leitch LC, Baker BE, Brickman L (1945). Synthesis of Sulphanilylthiourea and Related Compounds. Can J Res Sec B:Chem Sci.

[50] Skehan P, Storeng R, Scudiero D, Monks A, McMahon J, Vistica D, Warren JT, Bokesch H, Kenney S, Boyd MR (1990). New Colorimetric Cytotoxicity Assay for Anticancer-Drug Screening. J Natl Cancer Inst.

